# An Isotopic Ratio Outlier Analysis Approach for Global Metabolomics of Biosynthetically Talented Actinomycetes

**DOI:** 10.3390/metabo9090181

**Published:** 2019-09-10

**Authors:** Jordan Carey, Thanh Nguyen, Jennifer Korchak, Christopher Beecher, Felice de Jong, Amy L. Lane

**Affiliations:** 1 Chemistry Department, University of North Florida, Jacksonville, FL 32224, USA; 2 IROA Technologies, Ann Arbor, MI 48105, USA

**Keywords:** natural product, secondary metabolite, actinomycete, isotopic ratio outlier analysis, global metabolomics, diketopiperazine, liquid chromatography-mass spectrometry, siderophore

## Abstract

Actinomycetes are powerhouses of natural product biosynthesis. Full realization of this biosynthetic potential requires approaches for recognizing novel metabolites and determining mediators of metabolite production. Herein, we develop an isotopic ratio outlier analysis (IROA) ultra-high performance liquid chromatography-mass spectrometry (UHPLC/MS) global metabolomics strategy for actinomycetes that facilitates recognition of novel metabolites and evaluation of production mediators. We demonstrate this approach by determining impacts of the iron chelator 2,2′-bipyridyl on the *Nocardiopsis dassonvillei* metabolome. Experimental and control cultures produced metabolites with isotopic carbon signatures that were distinct from corresponding “standard” culture metabolites, which were used as internal standards for LC/MS. This provided an isotopic MS peak pair for each metabolite, which revealed the number of carbon atoms and relative concentrations of metabolites and distinguished biosynthetic products from artifacts. Principal component analysis (PCA) and random forest (RF) differentiated bipyridyl-treated samples from controls. RF mean decrease accuracy (MDA) values supported perturbation of metabolites from multiple amino acid pathways and novel natural products. Evaluation of bipyridyl impacts on the nocazine/XR334 diketopiperazine (DKP) pathway revealed upregulation of amino acid precursors and downregulation of late stage intermediates and products. These results establish IROA as a tool in the actinomycete natural product chemistry arsenal and support broad metabolic consequences of bipyridyl.

## 1. Introduction

Bacteria from the order Actinomycetales, commonly known as actinomycetes, are diverse and widespread across marine, freshwater, and terrestrial habitats [1]. Actinomycetes are prolific producers of small molecules, accounting for over 75% of bioactive bacterial natural products identified to date [2]. These structurally diverse metabolites serve as lead compounds for pharmaceutical and agrochemical development [3], inspire organic syntheses [4], mediate intra- and interspecies interactions [5], and provide chemical probes for elucidating mechanisms of biological processes [6].

While actinomycete metabolism has been intensely investigated for over 75 years [2], bioinformatics analyses support that actinomycete genomes encode a treasure trove of biosynthetic pathways with the potential of yielding as-yet unidentified secondary metabolites [7]. The unveiling of these metabolites poses substantial challenges, largely due to the complexity of actinomycete metabolomes [8], limited understanding of pathway regulation and triggers of pathway expression [9], low natural product yields [10], and repeated discovery of known metabolites [11]. These struggles have been tackled by approaches that include supplementation of cultures with small molecules hypothesized to mediate biosynthesis [12,13], co-cultivation of microorganisms to elicit metabolite production [12,14,15,16], heterologous expression of targeted biosynthetic pathways in hosts optimized for secondary metabolism [17,18], and creation of biosynthetic gene elimination mutants to facilitate recognition of metabolites encoded by these genes [18].

A variety of comparative bioactivity [14], gene transcript [12], protein [12], and metabolite profiling techniques [12,19] have been developed to evaluate changes in bacterial secondary metabolic pathway activity in response to experimental treatments. Innovative mass spectrometry (MS)-based metabolomics approaches have demonstrated particular promise in these efforts [8,20,21,22,23]. These approaches include imaging MS to pinpoint producers of novel metabolites during microbial co-cultivation [15,24,25] and liquid chromatography-mass spectrometry principal component analysis (LC/MS-PCA) metabolomics to efficiently prioritize strains with greatest potential for production of novel metabolites and evaluate impacts of co-cultivation [14,16,26,27]. Additionally, LC/MS with molecular networking by tandem MS has facilitated metabolite dereplication and recognition of unique molecular families [28,29].

Challenges of bacterial MS metabolomics include the differentiation of biosynthetic compounds from “red herring” artifacts introduced during chemical extraction or analysis, large numbers of candidate molecular formulae supported by exact mass data, and the need for many replicate samples to achieve statistically significant comparisons of metabolite abundances between experimental groups without use of internal standards [8,20,21,22,23]. Isotopic ratio outlier analysis (IROA) global metabolomics offers potential solutions to these bacterial metabolomics hurdles [30]. IROA LC/MS metabolomics was developed using *Caenorhabditis elegans* worm models [31,32], and IROA was recently adapted for gas chromatography-mass spectrometry (GC/MS) with *Saccharomyces cerevisiae* yeast models [33,34]. In these seminal IROA studies, treatment and control organisms were grown in media composed of uniformly 95:5 ^12^C:^13^C- and 5:95 ^12^C:^13^C-labeled carbon sources, respectively. Paired treatment and control organisms were pooled prior to chemical extraction and LC/MS or GC/MS. MS revealed an isotopic peak pair for each metabolite found in both treatment and control, and statistical comparison of peak areas between these isotopic partners using an automated pipeline rapidly pinpointed metabolites whose abundance differed between groups.

Using the IROA approach, metabolites were readily distinguished from artifacts based on distinctive isotopic peak pairs exclusive to biosynthetic products. Evaluation of the molecular ion *m*/*z* difference between ^12^C and ^13^C monoisotopomers readily revealed the number of carbon atoms in each metabolite, dramatically narrowing the number of molecular formulae feasible from exact masses. Further, the pooling of isotopically distinguishable metabolites from control and experimental samples prior to chemical extraction and analysis reduced variability associated with these steps, lowering the number of replicates required for comparison of metabolite abundances. Together, these features suggest the potential of IROA metabolomics as a tool for study of actinomycetes and other rich sources of natural products.

Herein, we establish an IROA UHPLC/MS global metabolomics approach for bacteria by evaluating responses of model actinomycete *Nocardiopsis dassonvillei* DSM 43111 to the iron chelator 2,2′-bipyridyl. Iron has previously been demonstrated as a mediator of natural product biosynthetic pathway activity in bacteria [35], and supplementation of media with bipyridyl, as a treatment, was previously used in other actinomycetes to emulate competition with iron-chelating bacteria and trigger the production of siderophores [24]. *N. dassonvillei* features rich secondary metabolic potential, with its genome encoding over a dozen natural product pathways predicted to yield metabolites from classes including polyketides, nonribosomal peptides, siderophores, and terpenoids [36,37]. One of these pathways was previously demonstrated to yield the nocazine/XR334 family of diketopiperazine (DKP) natural products [38], while metabolites corresponding to the other pathways remain cryptic. Our results illustrate the potential of IROA LC/MS metabolomics as a tool in the arsenal for realizing the biosynthetic potential of actinomycetes and highlight broad impacts of bipyridyl on *N. dassonvillei* metabolism.

## 2. Results

### 2.1. IROA Approach Enabling Detection of Metabolites Whose Production Is Initiated or Terminated in Response to Experimental Treatment

In published IROA metabolomics protocols [32,33,34], organisms from experimental and control groups were grown in media containing uniformly labeled carbon sources with different ^12^C:^13^C ratios (i.e., 95:5 vs. 5:95) and pooled prior to chemical extraction and UHPLC/MS or GC/MS. Comparison of the relative abundances of ^12^C- and ^13^C-labeled signals from isotopic peak pairs revealed differences in metabolite abundances between groups. This approach could not be used to find compounds that were absent from the experimental or control group, since a ^12^C- and ^13^C-labeled isotope peak pair would not exist. This limited applications of the existing IROA approach for natural product studies.

**Figure 1:**
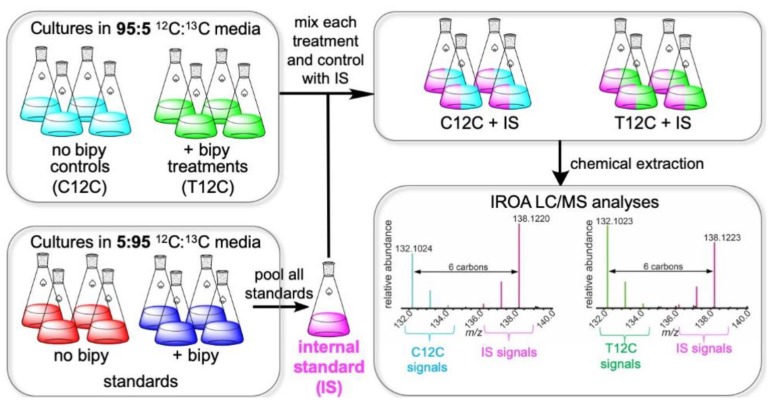
Overview of isotopic ratio outlier analysis (IROA) experimental approach for detecting metabolites whose production is upregulated, downregulated, or exclusive to treatments or controls. This approach was used to evaluate impacts of 2,2′-bipyridyl (bipy) on the *N. dassonvillei* metabolome. Bipyridyl treatment (*n* = 4, T12C) and no-bipyridyl control (*n* = 4, C12C) bacteria were fermented in uniformly 95:5 ^12^C:^13^C-labeled media alongside equivalent standard cultures in 5:95 ^12^C:^13^C-labeled media. Standard cultures were then pooled to provide an internal standard (IS) mixture containing ^13^C-labeled isotopes of every metabolite from T12C or C12C. Each T12C and C12C culture was supplemented with this IS before chemical extraction and UHPLC/MS. The ratio of C12C (blue) or T12C (green) MS signal integrals to corresponding IS signals (magenta) was determined. Statistical comparison of these C12C:IS and T12C:IS ratios enabled determination of metabolites whose production was impacted by bipyridyl treatment. Evaluation of the *m*/*z* difference between monoisotopic ^12^C- and ^13^C-labeled metabolites indicated the number of carbon atoms in each molecule, as illustrated by the IROA peak pair for leucine from *N. dassonvillei*.

Thus, we aimed to develop an IROA method that enabled the measurement of all metabolic changes, including metabolites whose production was initiated or terminated in response to experimental treatment. This key change required making a biochemically complex internal standard (IS) containing distinctive isotopes of all metabolites from every experimental or control group. This IS was prepared by fermenting both experimental (with bipyridyl) and control (without bipyridyl) *N. dassonvillei* in uniformly 5:95 ^12^C:^13^C-labeled media. These cultures were pooled at the conclusion of fermentation to provide a complete set of ^13^C-enriched metabolites for use as an IS (Figure 1). This IS was added to each treatment and control sample (T12C and C12C, respectively), which contained 95:5 ^12^C:^13^C-labeled metabolites, immediately prior to chemical extraction and metabolomics analyses. Thus, this approach provided an internal standard (IS) containing a unique isotope of all of the compounds in any of the experimental or control groups. Ratios of MS peak areas were determined for each compound from T12C or C12C relative to its isotopic IS using ClusterFinder software. When either T12C or C12C integrals were below the limit of quantitation, missing data points resulted. To facilitate statistical analyses, these data points were imputed by ClusterFinder algorithms. Statistical comparisons of T12C:IS vs. C12C:IS peak pair ratios for all metabolites then enabled evaluation of metabolites whose production was initiated, terminated, up-, or downregulated in response to bipyridyl treatment.

### 2.2. N. dassonvillei Metabolome Revealed by Untargeted IROA UHPLC/MS

To determine the feasibility of our IROA LC/MS approach for untargeted metabolomics of actinomycetes (Figure 1), metabolic changes resulting from treatment of the model actinomycete *N. dassonvillei* with bipyridyl were assessed. Evaluation of high-resolution electrospray ionization positive (ESI^+^) and ESI^−^ UHPLC/MS data for chemical extracts of bipyridyl treatment (T12C) and no bipyridyl control (C12C) cultures, each supplemented with IS, revealed 1332 T12C-IS and/or C12C-IS MS peak pairs (Table 1; Table S1). A variety of metabolites were detected exclusively from one sample type or differed dramatically between types (Table S2), supporting that bipyridyl treatment results in initiation, termination, upregulation, and downregulation of biosynthetic pathways over the evaluated fermentation period.

Computation of the difference in molecular ion *m*/*z*’s between ^12^C and ^13^C monoisotopic peak pair signals indicated that the number of carbon atoms in detected metabolites ranged from two to 36 (Supplementary Materials Figure S1). Of the 1332 metabolites supported by IROA peak pair analyses, identities of 107 molecules (Table S1) were supported by correspondence between number of carbon atoms, exact masses of molecular ions, and retention times with a library of standard compounds. Identified metabolites encompassed common classes of biomolecules including amino acids, dipeptides, carbohydrates, and metabolites from nucleic acid pathways (Table S1). For the majority of other metabolites, identifications were made to at least the level of molecular formula. This IROA technique excluded all non-biological signals from consideration, thereby assuring that all detected analytes truly represented *N. dassonvillei* metabolites rather than artifacts. Together, these data support the suitability of IROA approaches for evaluating actinomycete metabolomes and detecting a broad range of metabolites from this biosynthetically talented group of bacteria.

### 2.3. IROA LC/MS Differentiates Treatment and Control N. dassonvillei Metabolomes and Suggests the Production of Novel Metabolites

For each IROA MS peak pair detected from *N. dassonvillei* (Supplementary Materials Table S1), the ratio between signals from controls (C12C, *n* = 4) or treatments (T12C, *n* = 4) and IS was determined (Table S2). Univariate statistical comparison of these C12C:IS and T12C:IS ratios by unpaired two sample *t*-tests revealed that abundances of 253 metabolites were altered in response to bipyridyl treatment with *p* < 0.001. These metabolites (Table S3) were associated with a variety of primary metabolic pathways, including nucleic acid and amino acid pathways.

**Figure 2:**
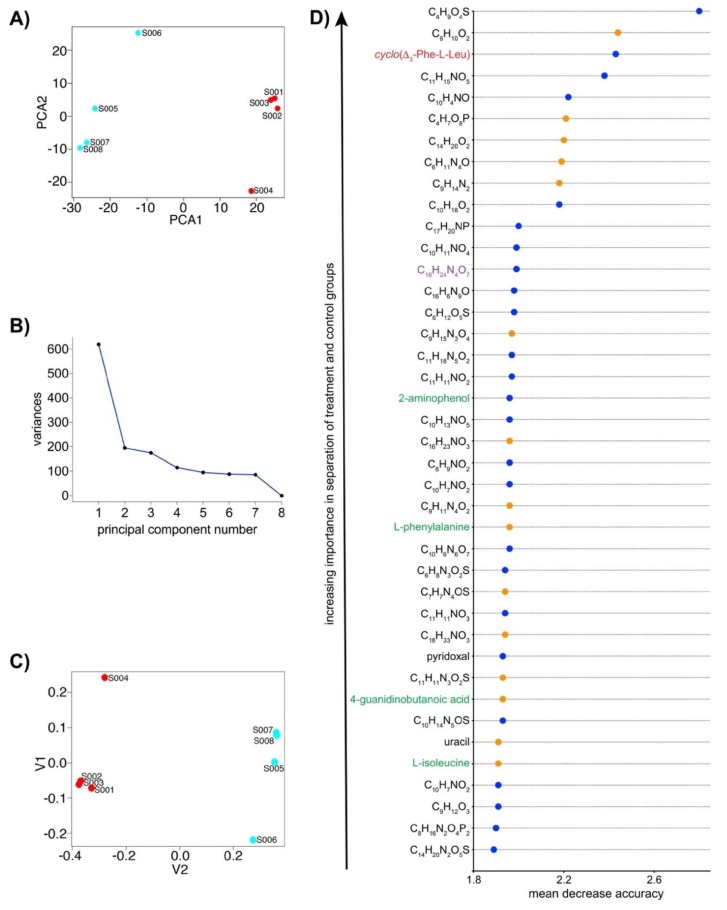
Multivariate analyses of bipyridyl treatment and no-bipyridyl control *N. dassonvillei* metabolomes. (**A**) A two-dimensional principal component analysis (PCA) score plot supported impacts of bipyridyl on the *N. dassonvillei* metabolome. Data points corresponding to controls are in red (S001–S004) and are differentiated from light blue data points corresponding to treatments (S005–S008). (**B**) A scree plot of PCA component variances supported that PC1–PC2 account for most of the deviation between treatment and control groups. (**C**) A multidimensional scaling plot of proximity matrix from random forest (RF) segregated treatment and control samples. X- and y-axes indicate multidimensional scaling coordinates, and data points are colored as in (**A**). (**D**) RF variable importance plot of 40 metabolites contributing most toward RF separation of groups. Annotated metabolites associated with amino acid metabolism, based on the KEGG database, are named in green. Previously reported secondary metabolites are named in red, and novel putative molecular formulae with >10 C are in purple. Blue data points indicate metabolites that decreased in response to bipyridyl, and orange indicates metabolites whose abundance increased in response to this iron chelator.

To further evaluate metabolic perturbation resulting from bipyridyl treatment, both supervised and unsupervised multivariate analyses were conducted (Figure 2). Principal component analysis (PCA), an unsupervised method [39], was employed to provide a global overview of variance between treatment and control groups. PCA clearly distinguished between samples from these two groups (Figure 2A), supporting perturbation of the *N. dassonvillei* metabolome in response to bipyridyl. The PCA scree plot (Figure 2B) revealed that the first two PCs account for most of the variation between *N. dassonvillei* treatment and control groups.

Random forest (RF) analysis, a supervised multivariate method [40], was conducted to further evaluate variance between treatment and control groups and to determine individual metabolites most important for distinguishing between groups. RF differentiated all treatments from controls (Figure 2C), and the mean decrease accuracy (MDA) was determined for individual metabolites (Figure 2D). MDA measures the decrease in performance of an RF model if an individual metabolite was removed from the analysis [40]. Hence, a larger MDA value corresponds to greater importance of a metabolite in predicting the sample group and provides evidence for metabolic signatures associated with experimental treatments. The 40 *N. dassonvillei* metabolites with highest MDA scores included molecules up- and downregulated in response to bipyridyl treatment and spanned multiple classes of primary metabolites (Figure 2D, Figure S2, Table S4). Molecules from amino acid pathways (i.e., l-phenylalanine, 4-guanidinobutanoic acid, 2-aminobenzoic acid, l-isoleucine) were prominent among metabolites with largest MDA values. The metabolic signature associated with bipyridyl treatment also included a variety of as-yet unidentified metabolites. Molecular formulae of these metabolites were predicted based on the IROA-supported number of carbon atoms along with correspondence between theoretical and experimental exact masses for molecular ions (Figure 2D).

The MDA-supported molecular signature (Figure 2D) was further evaluated for potentially novel secondary metabolites. Since most recently discovered bioactive natural products possess >10 carbon atoms, we focused on metabolites within this carbon number range. An IROA-supported carbon number of 16 together with experimental [M + H]^+^ *m*/*z* 385.1761 supported C_16_H_24_N_4_O_7_ as a putative molecular formula (Figure S3) that, to our knowledge, is unprecedented among bacterial natural products. Unfortunately, fermentation at an eight-liter scale failed to yield this metabolite in quantities sufficient for structure elucidation.

### 2.4. Impacts of Bipyridyl Treatment on N. dassonvillei Biomass and Siderophore Production

**Figure 3:**
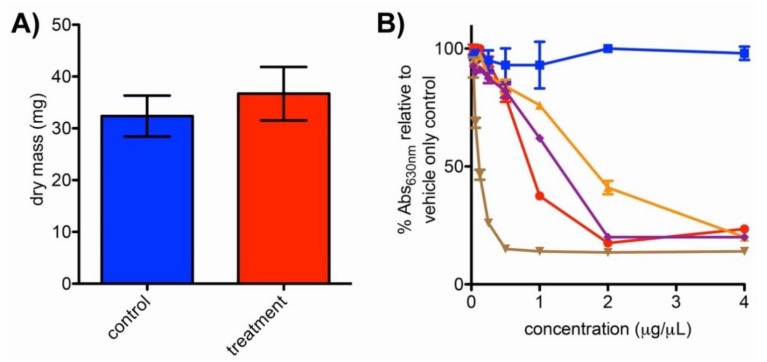
Evaluation of *N. dassonvillei* biomass and siderophore titer changes in response to fermentation in bipyridyl-containing media for seven days. (**A**) Dry mass of cell pellets from *N. dassonvillei* treatments fermented in 50 mL media with bipyridyl relative to controls fermented without bipyridyl. An unpaired *t*-test (*n* = 4) revealed *p* = 0.23. (**B**) Comparison of siderophore activity across chemical extracts from *N. dassonvillei* culture types and media only controls. Decreased absorbance at 630 nm corresponds to increased siderophore concentration, as illustrated by desferrioxamine B positive controls (brown triangles). Blue squares denote activity of extracts from control *N. dassonvillei* grown in media without bipyridyl, while red circles indicate activity of *N. dassonvillei* treatments fermented with bipyridyl. These treatment extracts were markedly more active than those from bipyridyl-containing media only controls (orange triangles), supporting that *N. dassonvillei* produces siderophores. Comparable activities were observed for treatment extracts (red circles) and extracts formed by mixing extracts from bipyridyl-containing media with bipyridyl-free *N. dassonvillei* cultures (purple diamonds), suggesting that bipyridyl treatment does not dramatically upregulate siderophore production. Error bars indicate standard deviation for *n* = 4 replicates.

To evaluate whether observed metabolic changes (Figure 2) may relate to decreased growth in bipyridyl-treated *N. dassonvillei* cultures, biomasses of cell pellets from treatments and controls (*n* = 4) were determined at the conclusion of fermentation. This revealed no significant difference (unpaired *t*-test, *p* = 0.23) in dry mass resulting from fermentation in the presence or absence of bipyridyl (Figure 3A).

To evaluate siderophore production as a potential mechanism by which *N. dassonvillei* may mitigate impacts of iron limitation, ethyl acetate chemical extracts from treatment and control cultures were subjected to an established chemical assay for siderophore detection [41]. Activity in this assay was greater for extracts from *N. dassonvillei* grown in media supplemented with bipyridyl than for control chemical extracts of bipyridyl-supplemented media (Figure 3B), supporting the production of siderophores by bipyridyl-treated *N. dassonvillei.* In contrast, extracts from *N. dassonvillei* grown in media without bipyridyl were not active at evaluated concentrations. This difference in activity between *N. dassonvillei* sample types suggested that bipyridyl treatment upregulates siderophore production, or alternatively, that activity is enhanced by interactions between constitutively produced siderophores and bipyridyl extracted from the media. To evaluate these possibilities, the activity of extracts from *N. dassonvillei* grown in bipyridyl-containing media was compared with mixtures of extracts from bipyridyl-containing media and *N. dassonvillei* fermented in the absence of bipyridyl. Comparable activities were observed between these two sample types (Figure 3B), suggesting that siderophore assay activity results primarily from additive effects of bipyridyl with constitutive *N. dassonvillei* siderophores rather than from dramatic upregulation of siderophore biosynthesis.

### 2.5. IROA Enables Rapid Assessment of the Perturbation of Targeted Metabolic Pathways

**Figure 4:**
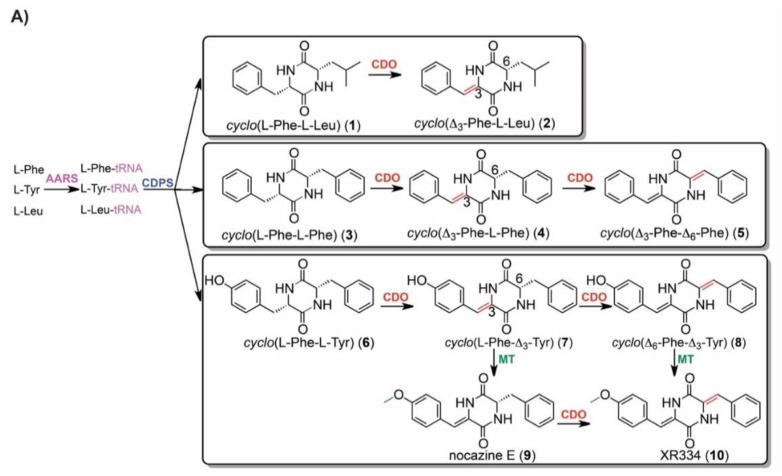

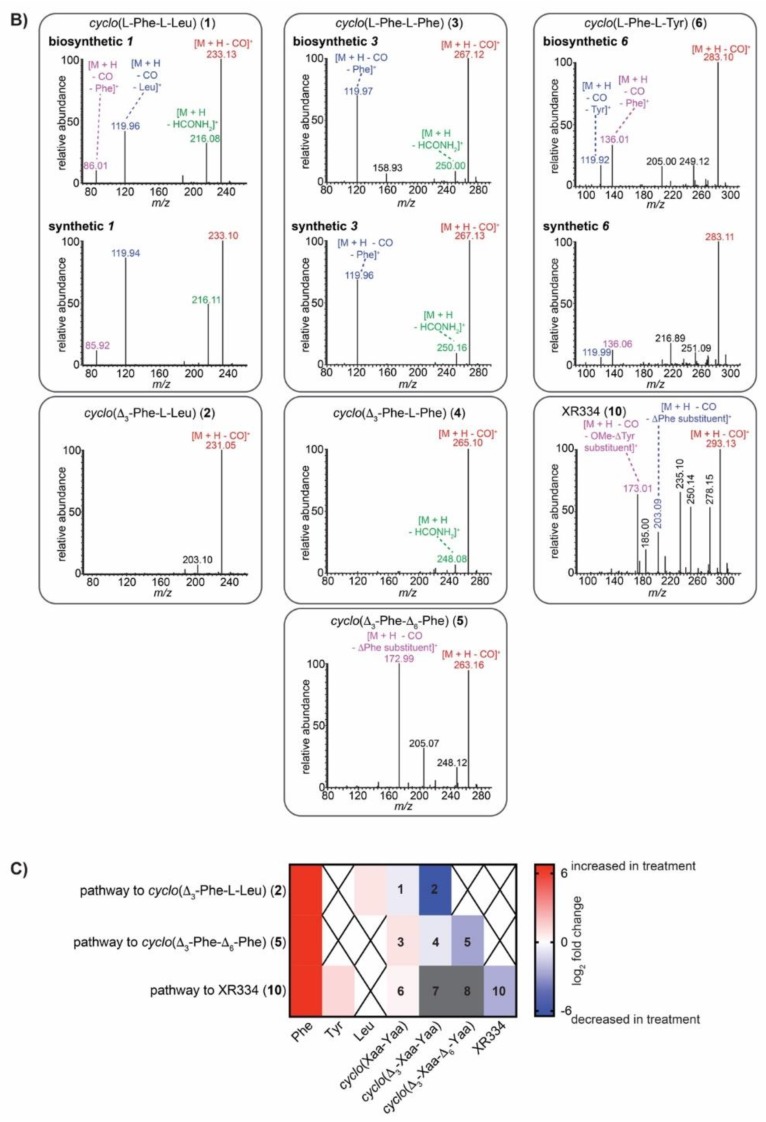
Application of IROA metabolomics for evaluating impacts of small molecules on targeted natural product biosynthetic pathways. Impacts of bipyridyl on *N. dassonvillei* production of DKPs 1–10 were evaluated. (**A**) The previously reported biosynthetic pathway for 1–10 includes aminoacyl tRNA synthetase (AARS)-catalyzed ligation of amino acids with tRNAs, followed by cyclodipeptide synthase (CDPS)-catalyzed formation of DKPs 1, 3, and 6 from different combinations of aminoacyl-tRNAs. Tailoring of these DKP intermediates by a cyclic dipeptide oxidase (CDO) and/or methyltransferase (MT) leads to final products 2, 5, and 10 [38]. (**B**) LC/MS^2^ supported identification of each DKP from *N. dassonvillei* extracts. Comparison of MS^2^ fragments for 1, 3, and 6 from *N. dassonvillei* with synthetic standards supported their identification, while dehydrogenated and/or methylated derivatives were confirmed by correspondence between observed fragments shown in color with literature values [38,42]. (**C**) Heatmap of mean (*n* = 4) log_2_ fold changes in abundance of DKP pathway metabolites in response to bipyridyl. From left-to-right, the heatmap is arranged in the order of precursors leading to final DKPs 2, 5, and 10. Map fields marked with “X” indicate metabolites not required for production of the final DKP indicated in that row, fields in gray indicate previously reported pathway intermediates that were not detected in our experiments, and numbers superimposed within the heatmap correspond to compound numbers from (**A**). Xaa and Yaa indicate Phe, Leu, or Tyr residues of DKPs.

The optimization of high-value secondary metabolic pathways requires understanding of the impacts of experimental variables on pathway precursors, intermediates, and products. Aiming to establish IROA as a tool for evaluating mediators of targeted pathways, we assessed the effects of bipyridyl on the previously reported nocazine/XR334 diketopiperazine (DKP) biosynthetic pathway from *N. dassonvillei* [38] (Figure 4A). Production of nocazine/XR334 pathway final products (i.e., **2**, **5**, **10**) is catalyzed by a suite of promiscuous enzymes, with these DKP products assembled from different amino acid precursors and differing in methylation and/or dehydrogenation tailoring (Figure 4A).

Inspection of the *N. dassonvillei* IROA dataset revealed MS exact masses and carbon numbers suggesting DKPs 1–6 and 10 and amino acid precursors leucine, phenylalanine, and tyrosine (Table S1; Figure S4). MS signals indicative of 7–9 were not observed. The identity of each amino acid precursor was confirmed by comparison of LC retention time and MS exact mass with authentic standards. Structures of DKPs 1–6 and 10 were confirmed by correspondence between tandem mass spectra (Figure 4B) of *N. dassonvillei* metabolites with commercial standards and/or literature values [38,42]. Intriguingly, identification of these metabolites revealed that *cyclo*(Δ_3_-Phe-L-Leu) 2 was among those metabolites with greatest MDAs (Figure 2D).

The abundance of each nocazine/XR334 pathway precursor, intermediate, and product (Figure 4a) was compared between bipyridyl treatment (*n* = 4) and no bipyridyl control (*n* = 4) *N. dassonvillei* cultures. A similar trend was observed along the pathways leading to final products **2**, **5**, and **10**. For each, amino acid precursors were upregulated in treatments, while downregulation of late stage intermediates and products was observed (Figure 4C). This finding supports that bipyridyl perturbs the nocazine/XR334 pathway and suggests a particularly negative impact on later stages of the pathway. It also highlights the promise of IROA metabolomics for efficiently tracking impacts of experimental variables, such as small molecules, on targeted secondary metabolic pathways.

## 3. Discussion

Our work provides the first demonstration of IROA LC/MS metabolomics for actinomycetes, a biotechnologically important group of bacteria renowned as secondary metabolite producers [1,2,7]. It also establishes an IROA protocol (Figure 1) that enables recognition of metabolites which are exclusive to some experimental groups. This opens doors for new applications of IROA, such as the tracking of metabolites corresponding to cryptic biosynthetic pathways via comparison of metabolomes between wild type and biosynthetic gene elimination mutant organisms. Likewise, microbial co-cultivation and exposure to small molecules have previously been demonstrated to initiate secondary metabolite production [12,13,14,15,16,24], and the IROA protocol herein provides a novel strategy for recognition of these induced natural products.

The IROA approach offers several features that complement previously developed strategies for MS-based metabolomics of actinomycetes. The ^12^C/^13^C isotopic peak pair observed for each metabolite not only enables comparison of metabolite abundances across experimental and control groups, but also differentiates artifact signals from biosynthetic products and reveals the number of carbon atoms in each metabolite [32]. Determination of the number of carbon atoms in combination with high resolution exact masses dramatically reduces the number of plausible molecular formulae relative to conventional MS approaches [32], facilitating recognition of metabolites with novel molecular formulae. This was highlighted by the metabolic signature resulting from bipyridyl treatment (Figure 2d), which included a metabolite with a putative molecular formula of C_16_H_24_N_4_O_7_ that, to our knowledge, is unprecedented among bacterial natural products. IROA recognition of the number of carbon atoms also facilitates identification of previously characterized metabolites, as demonstrated herein for the nocazine/XR334 family of natural products (Figure 4; Figure S4).

Our findings highlight the utility of IROA metabolomics for global evaluation of changes to both primary and secondary metabolism. PCA and RF, complementary multivariate analysis methods [39,40], each clearly differentiated metabolomes from bipyridyl-treated and no-bipyridyl control *N. dassonvillei* in our study and suggest broad-reaching metabolic consequences of bipyridyl (Figure 2). The large number of metabolic changes observed in our study creates challenges for prioritization of specific metabolites for identification. Future integration of IROA approaches with burgeoning MS-based molecular networking approaches [28,29] and actinomycete-specific secondary metabolite databases [43] is anticipated to promote dereplication of known metabolites and facilitate prioritization of promising novel metabolites.

Metabolome changes observed in the current study may result from direct responses of *N. dassonvillei* to bipyridyl or, more likely, from responses to diminished free iron due to chelation by bipyridyl. While iron is required for a variety of metabolic processes, its availability in nature is limited by the low solubility of Fe^3+^ [44]. Bacteria have adapted to iron limitation in many ways, including the constitutive or inducible production of siderophores that sequester iron for the producing organism [44]. For example, Vinayavekhin et al. demonstrated that low iron levels stimulated production of a family of novel siderophoric 2-alkyl-4,5-dihydrothiazole-4-carboxylates by proteobacterium *Pseudomonas aeruginosa* [35]. Likewise, Traxler et al. used imaging mass spectrometry to reveal that interactions with siderophore-producing bacteria stimulated the production of novel siderophoric acylated desferrioxamines by *Streptomyces coelicolor*, presumably as an evolutionary strategy to compete for iron [24]. Our study suggests that *N. dassonvillei* produces siderophore(s) of as-yet unknown structures (Figure 3B). Siderophore production offers one plausible explanation of how *N. dassonvillei* may avert significant reductions in biomass in response to iron (III) limitation during bipyridyl treatment (Figure 3A).

While the *N. dassonvillei* genome encodes a pathway homologous to the desferrioxamine pathway from *S. coelicolor* (70–75% amino acid similarity) [36,37,45], this group of siderophores has not been previously reported from *N. dassonvillei* and our IROA analyses likewise revealed no evidence for production of known desferrioxamines. This suggests that the *N. dassonvillei* desferrioxamine pathway is silent or encodes novel desferrioxamines. The *N. dassonvillei* genome also encodes multiple cryptic nonribosomal peptide synthetase and polyketide synthase pathways, which potentially yield as-yet unidentified siderophores.

Together, our results illuminate impacts of bipyridyl on the metabolome of a model actinomycete and provide the first implementation of IROA global metabolomics for study of actinomycetes. Our results support IROA as a tool in the pipeline for natural product evaluation, suggesting its utility for recognition of metabolites with novel molecular formulae and for evaluating impacts of experimental treatments, such as small molecules, on primary and secondary metabolism.

## 4. Materials and Methods

### 4.1. Nocardiopsis dassonvillei Cultures and Fermentation for IROA Experiments

*Nocardiopsis dassonvillei* (Brocq-Rousseau 1904) Meyer 1976 DSM 43111 (ATCC 23218) was obtained from the American Type Culture Collection and maintained on ISP-2 agar plates. *N. dassonvillei* experimental and control cultures were prepared using media composed of 1.5 g protein hydrolysate, 0.8 g yeast extract, and 1 g glucose per L of deionized water, with each carbon source uniformly 95:5 ^12^C:^13^C-labeled (IROA Technologies). Treatment cultures (3 mL, *n* = 4) were supplemented with 2,2′-bipyridyl (200 μM, Sigma Aldrich) in DMSO vehicle, while control cultures (3 mL, *n* = 4) were supplemented with an equal amount of DMSO without bipyridyl. Additionally, standard cultures were prepared using the same media formulations, methods, and number of cultures as above, but containing uniformly 5:95 ^12^C:^13^C-labeled carbon sources. Cultures for tandem MS were prepared as above, using media without isotope labels. All cultures were fermented at 30 °C with shaking at 220 rpm for 7 days prior to chemical extraction.

### 4.2. Chemical Extraction and Preparation of Samples for UHPLC/MS Metabolomics

Following fermentation, all four replicate standard cultures were pooled to yield a 5:95 ^12^C:^13^C-labeled metabolite mixture for use as an IS. Each treatment and control culture was supplemented (1:1) with this IS, extracted twice with an equal volume of ethyl acetate, and solvent removed in vacuo. Chemical extracts were resolubilized with 500 μL of methanol for UHPLC/MS.

### 4.3. UHPLC/MS Analysis

Reversed phase UHPLC/MS was conducted using a Dionex Ultimate 3000 UHPLC pump interfaced with a Thermo Fisher Q-Exactive Orbitrap mass spectrometer. An ACE Excel PFP-C18 2.1 × 100 mm with 2.0 μm particle size column (ACE #EXL-1010-1002U) was used, with 2 μL injections of chemical extracts. UHPLC was conducted using a flow rate of 350 μL/min with water and acetonitrile gradient, where each contained 0.1% formic acid. An initial mobile phase of 100% water was held for 3 min, followed by a linear gradient from 0–80% acetonitrile over 10 min, then held at 80% acetonitrile for 3 min. MS was conducted in ESI^+^ and ESI^−^ modes with a probe temperature of 350 °C, spray voltage of 3500 V, capillary temperature of 320 °C, sheath gas flow rate of 40 arbitrary units, and auxiliary gas flow rate of 10 arbitrary units. Tandem mass spectra for DKPs 1–6 and 10 were collected using a Dionex Ultimate 3000 UHPLC pump interfaced with a Thermo Fisher LTQ-XL mass spectrometer in ESI^+^ mode with parameters analogous to those above and a normalized collision energy of 40 for MS^2^.

### 4.4. Data Processing and Analyses

Thermo raw datafiles were converted into mzxml files and imported into IROA ClusterFinder software V3.0 (IROA Technologies) for analysis. ClusterFinder was used following manufacturer protocols for automated recognition of IROA peak pairs and prediction of molecular formulae, followed by statistical comparisons of abundances of all recognized ^12^C- and ^13^C-labeled isotopic peak pairs. PCA and RF analysis, including MDA evaluation, were conducted using the Statistical Tools feature of ClusterFinder. Indicated primary metabolites were identified by comparison of retention time, IROA-predicted molecular formula, and molecular ion *m*/*z* with the Mass Spectrometry Metabolite Library of Standards (MSMLS, IROA Technologies). Plausible identities of other metabolites were proposed based on comparison of predicted molecular formula with molecules from the ClusterFinder database (IROA Technologies). Box-whisker plots and heatmaps, as well as statistics associated with these plots, were prepared using GraphPad Prism 8.

### 4.5. Evaluation of Biomass and Siderophore Production

Treatment (with bipyridyl) and control (no bipyridyl) cultures (*n* = 4) were prepared at 150 mL scales using fermentation parameters and media as above, but containing natural abundances of isotopes. At the conclusion of fermentation, cell pellets were prepared from 50 mL of each culture by centrifugation at 5000 rpm for 15 min, pellets washed twice with DI water, and dried at 95 °C for 48 h before determination of dry mass. Statistical comparisons of dry masses were conducted using GraphPad Prism 5 with an unpaired t-test.

From each culture, 100 mL was extracted twice with an equal volume of ethyl acetate, and solvent removed in vacuo. Control extracts (*n* = 4) corresponding to media supplemented with bipyridyl (no bacteria) were prepared equivalently. Extracts were reconstituted with DMSO vehicle for siderophore assays.

Siderophore production was evaluated using an established chemical assay, in which Fe^3+^, chrome azurol S, and hexadecyltrimethylammonium bromide form a blue tertiary complex that exhibits strong Abs_630_. This complex is disrupted in proportion to siderophore concentration, with a corresponding Abs_630_ decrease [41]. Assays were conducted in 96-well plates at eight concentrations of each chemical extract in a 1:1 serial dilution series ranging from 4 to 0.03 μg/μL. Desferrioxamine B mesylate (MilliporeSigma) was evaluated at this concentration range as a positive control. Following incubation at room temperature until color change equilibrium was achieved (~4 h), siderophore activity was evaluated by comparing Abs_630_ values for all samples relative to corresponding DMSO vehicle-only negative controls. Standard deviations were computed and plotted using GraphPad Prism 5.

## Figures and Tables

**Table 1:** Summary of *N. dassonvillei* metabolome revealed by IROA UHPLC/MS. IROA peak pair refers to a pair of molecular ion signals corresponding to a ^12^C-labeled metabolite from T12C and/or C12C and its isotopic ^13^C-labeled IS. Metabolite identities were proposed from agreement between molecular ion exact masses, IROA-determined number of carbon atoms, and retention times with compounds from a standard library.

	ESI Mode		
	Positive	Negative	Total
IROA peak pairs detected	1243	89	1332
Identified metabolites	85	22	107

## Supplementary Materials

The following are available online at https://www.mdpi.com/2218-1989/9/9/181/s1, Microsoft Excel spreadsheet containing Table S1: Summary of metabolites detected by IROA; Table S2: Isotopic ratios for all metabolites; Table S3: Metabolites deemed significant by univariate statistical analysis. Supplementary information PDF containing Figure S1: Histogram of number of carbon atoms in metabolites; Figure S2: Box-whisker plots of 40 metabolites with largest MDA; Figure S3: IROA molecular ion for putative novel C_16_ metabolite; Figure S4: IROA molecular ions for DKPs 1–6 and 10; Table S4: Summary of IROA LC/MS data for 40 metabolites with largest MDA.
