# Supplementary material for: An Isotopic Ratio Outlier Analysis Approach for Global Metabolomics of Biosynthetically Talented Actinomycetes
Source: Metabolites. 2019 Sep 10;9(9):181. doi: 10.3390/metabo9090181 (PMC6780544; doi:10.3390/metabo9090181)
Supplement: Supplementary file 1 [file metabolites-09-00181-s001.zip › supplementary materials/Supplementary Materials.pdf]

Supplementary Materials for An Isotopic Ratio Outlier Analysis Approach for Global Metabolomics of Biosynthetically Talented Actinomycetes

Jordan Carey[1], Thanh Nguyen[1], Jennifer Korchak[1], Christopher Beecher[2], Felice de Jong[2] and Amy L. Lane[1,*]

[1] Chemistry Department, University of North Florida, Jacksonville, FL 32224, USA; Email: n00666958@ospreys.unf.edu (J.C.); n00931601@ospreys.unf.edu (T.N.); n00958433@ospreys.unf.edu (J.K.)
[2] IROA Technologies, Ann Arbor, MI 48105, USA; Email: chris@iroatech.com (C.B.); felice@iroatech.com (F.d.J.)
* Correspondence: amy.lane@unf.edu; Tel.: +01-904-620-2315

Contents:

Page #	Item
2	Figure S1. Histogram of the number of carbon atoms from *N. dassonvillei* metabolites detected by IROA UHPLC/MS.
3-6	Figure S2. Box-whisker plots of relative abundances for 40 metabolites with largest mean decrease accuracy (MDA).
7	Figure S3. IROA molecular ion peak pair for putative novel $C_{16}H_{24}N_4O_7$ metabolite from *N. dassonvillei*.
8-10	Figure S4. IROA molecular ion peak pairs for DKPs **1-6** and **10** from *N. dassonvillei*.
11	Table S4. Summary of IROA LC/MS data for 40 metabolites most strongly distinguishing *N. dassonvillei* bipyridyl treatment from no bipyridyl control groups based on RF MDA.

Note: Tables S1-S3 are provided as a standalone .xlsx file

Table S1. Summary of metabolites detected from *N. dassonvillei* chemical extracts by evaluation of UHPLC/MS IROA signals.

Table S2. Ratio of T12C:IS and C12C:IS MS signals for each metabolite detected from *N. dassonvillei* chemical extracts.

Table S3. *N. dassonvillei* metabolites for which univariate statistical comparison of C12C:IS and T12C:IS ratios by unpaired two sample t-test revealed $p < 0.001$.

Figure S1. Histogram of the number of carbon atoms from 1,332 *N. dassonvillei* metabolites detected by IROA UHPLC/MS. The number of carbon atoms in each metabolite was proposed by finding the *m/z* difference between the ^{12}C- and ^{13}C monoisotopic molecular ion signal for each analyte.

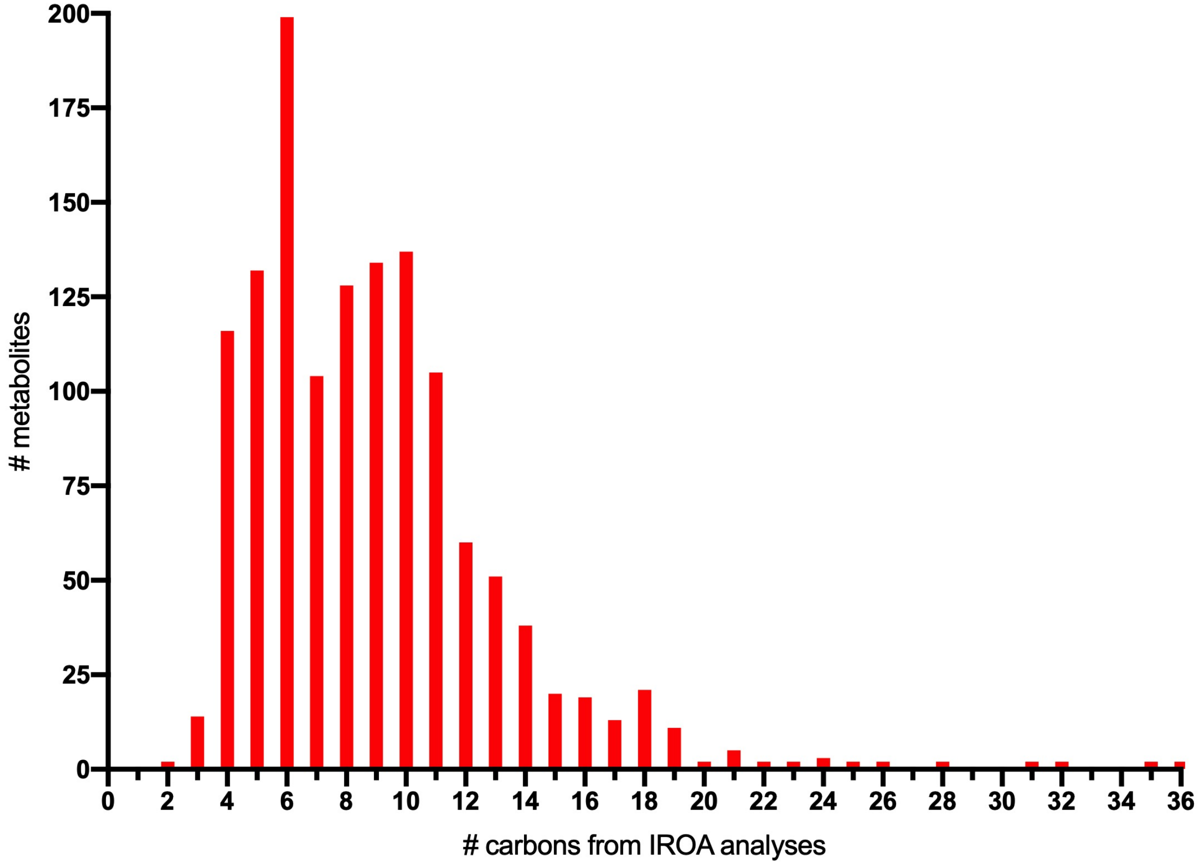

Figure S2. Box-whisker plots of relative abundances for 40 metabolites with largest mean decrease accuracy (MDA). MDA indicates which metabolites were most important in differentiating bipyridyl treatment (T12C) from no bipyridyl control (C12C) *N. dassonvillei* metabolomes in random forest (RF) analysis. Plots are shown in order of decreasing MDA; MDA values are in Fig. 2d of the main text and Table S4. Plots show the ratio of T12C:IS (n = 4, green) and C12C:IS (n = 4, blue) peak pairs. The horizontal line in box indicates the mean ratio, whiskers denote range from minimum to maximum, the top border of box indicates 75%th percentile, and bottom border of box denotes 25%th percentile ratio. The p-value for comparison of metabolite abundance between T12C and C12C was found using an unpaired t-test. Compound IDs (i.e. SECIM.xxx) correspond to metabolites summarized in Table S4.

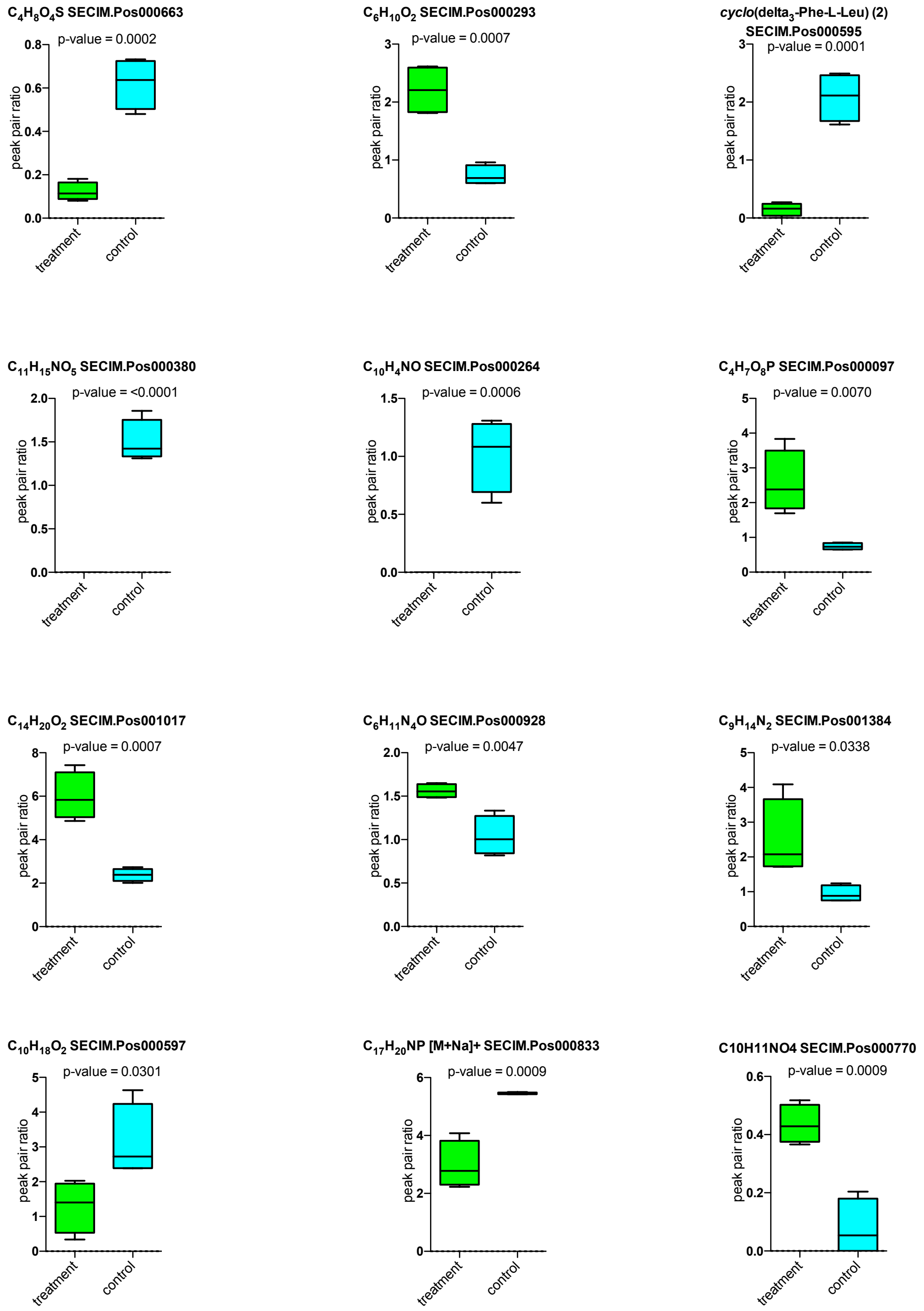

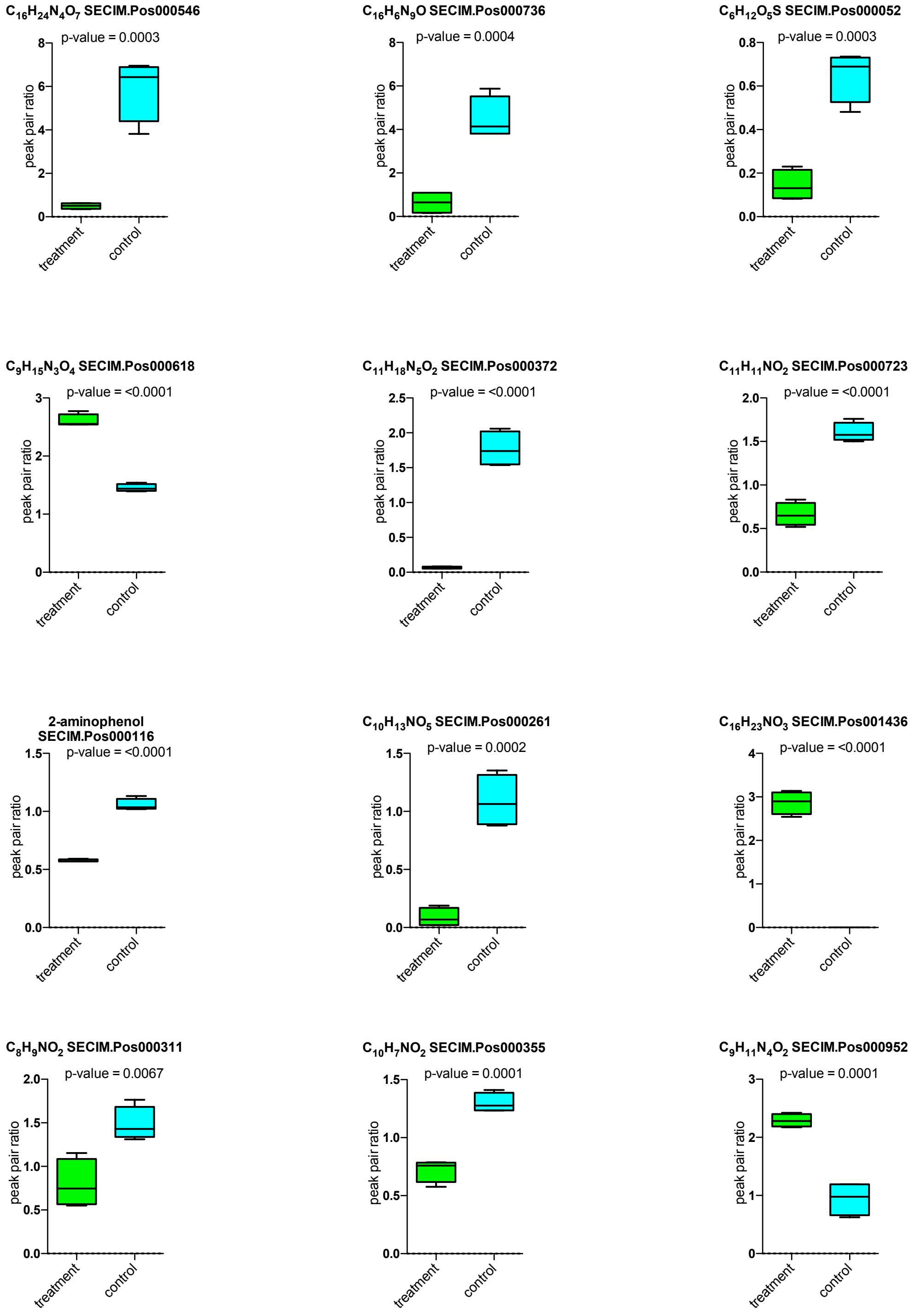
$C_{16}H_{24}N_4O_7$ SECIM.Pos000546
p-value = 0.0003
$C_{16}H_6N_9O$ SECIM.Pos000736
p-value = 0.0004
$C_6H_{12}O_5S$ SECIM.Pos000052
p-value = 0.0003
$C_9H_{15}N_3O_4$ SECIM.Pos000618
p-value = <0.0001
$C_{11}H_{18}N_5O_2$ SECIM.Pos000372
p-value = <0.0001
$C_{11}H_{11}NO_2$ SECIM.Pos000723
p-value = <0.0001
2-aminophenol SECIM.Pos000116
p-value = <0.0001
$C_{10}H_{13}NO_5$ SECIM.Pos000261
p-value = 0.0002
$C_{16}H_{23}NO_3$ SECIM.Pos001436
p-value = <0.0001
$C_8H_9NO_2$ SECIM.Pos000311
p-value = 0.0067
$C_{10}H_7NO_2$ SECIM.Pos000355
p-value = 0.0001
$C_9H_{11}N_4O_2$ SECIM.Pos000952
p-value = 0.0001
peak pair ratio
treatment
control

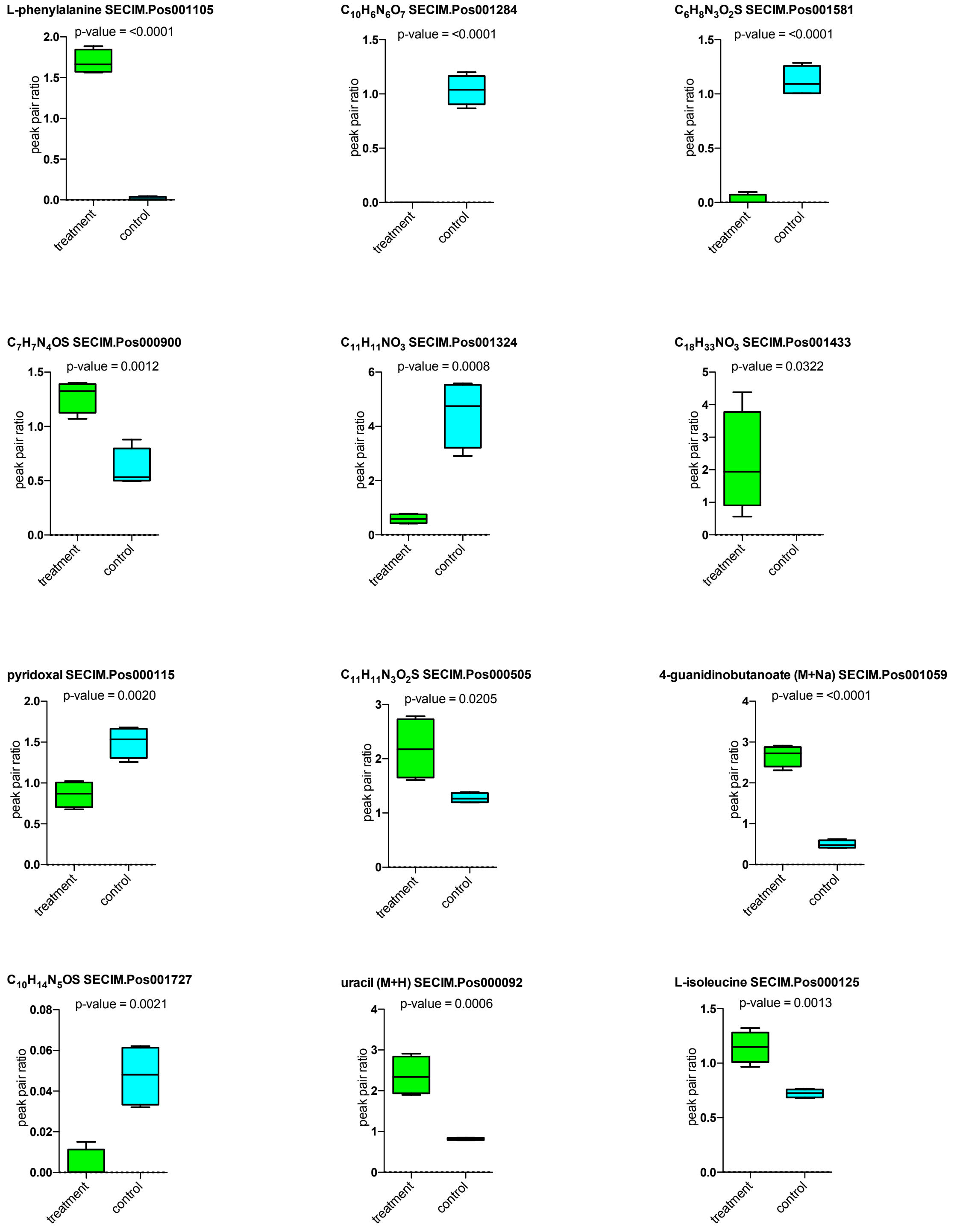
L-phenylalanine SECIM.Pos001105
p-value = <0.0001
$C_{10}H_6N_6O_7$ SECIM.Pos001284
p-value = <0.0001
$C_6H_8N_3O_2S$ SECIM.Pos001581
p-value = <0.0001
$C_7H_7N_4OS$ SECIM.Pos000900
p-value = 0.0012
$C_{11}H_{11}NO_3$ SECIM.Pos001324
p-value = 0.0008
$C_{18}H_{33}NO_3$ SECIM.Pos001433
p-value = 0.0322
pyridoxal SECIM.Pos000115
p-value = 0.0020
$C_{11}H_{11}N_3O_2S$ SECIM.Pos000505
p-value = 0.0205
4-guanidinobutanoate (M+Na) SECIM.Pos001059
p-value = <0.0001
$C_{10}H_{14}N_5OS$ SECIM.Pos001727
p-value = 0.0021
uracil (M+H) SECIM.Pos000092
p-value = 0.0006
L-isoleucine SECIM.Pos000125
p-value = 0.0013
peak pair ratio
treatment
control

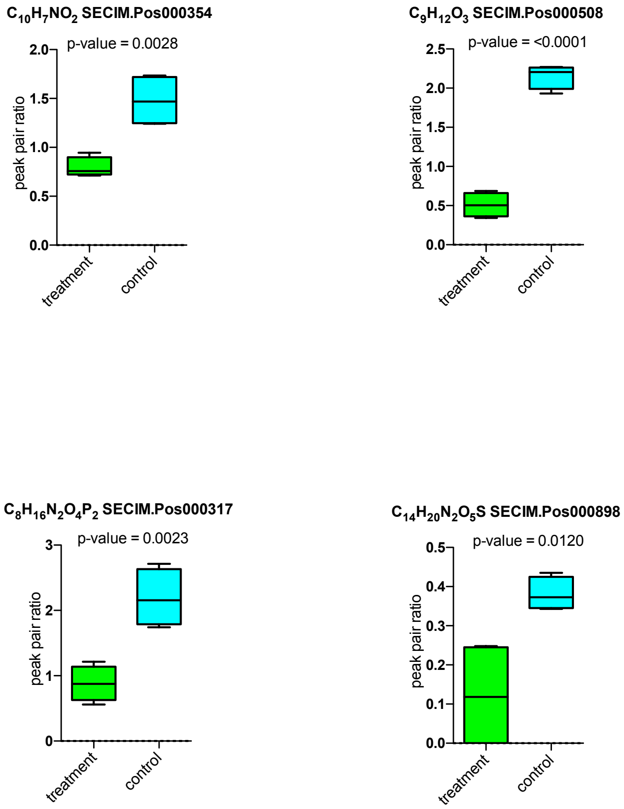
$C_{10}H_7NO_2$ SECIM.Pos000354
p-value = 0.0028
peak pair ratio
2.0
1.5
1.0
0.5
0.0
treatment
control
$C_9H_{12}O_3$ SECIM.Pos000508
p-value = <0.0001
peak pair ratio
2.5
2.0
1.5
1.0
0.5
0.0
treatment
control
$C_8H_{16}N_2O_4P_2$ SECIM.Pos000317
p-value = 0.0023
peak pair ratio
3
2
1
0
treatment
control
$C_{14}H_{20}N_2O_5S$ SECIM.Pos000898
p-value = 0.0120
peak pair ratio
0.5
0.4
0.3
0.2
0.1
0.0
treatment
control

Figure S3. IROA molecular ion peak pair for putative novel $C_{16}H_{24}N_4O_7$ metabolite from *N. dassonvillei*. The number of carbon atoms is indicated by a red arrow, and equals the *m/z* difference between the proposed $[M+H]^+$ signal for ^{12}C and ^{13}C monoisotopes; *m/z*'s comprising the IROA peak pair are indicated with enlarged and bolded font. The depicted mass spectrum is from *N. dassonvillei* control culture, was collected at a retention time of 9.8 min, and corresponds to compound ID SECIM.Pos000546 in Tables S1 and S4.

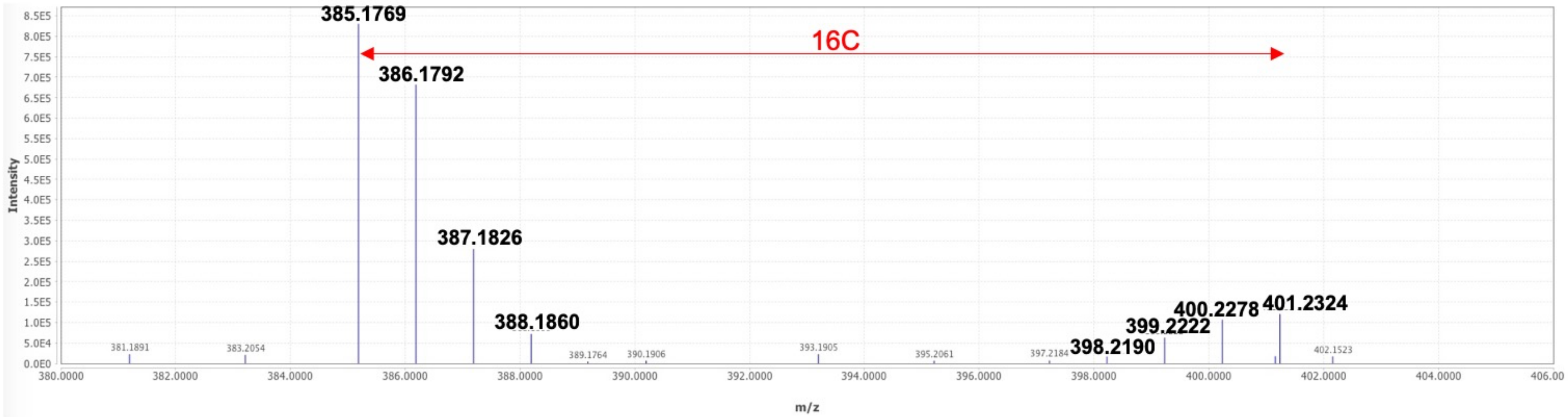

Figure S4. IROA molecular ion peak pairs for DKPs 1-6 and 10 from *N. dassonvillei*. The number of carbon atoms is indicated by a red arrow, and equals the *m/z* difference between the proposed $[M+H]^+$ signal for ^{12}C and ^{13}C monoisotopes; *m/z*'s comprising the IROA peak pair are indicated with enlarged and bolded font. RT indicates retention time at which each mass spectrum was collected. Data shown below were from *N. dassonvillei* control cultures.

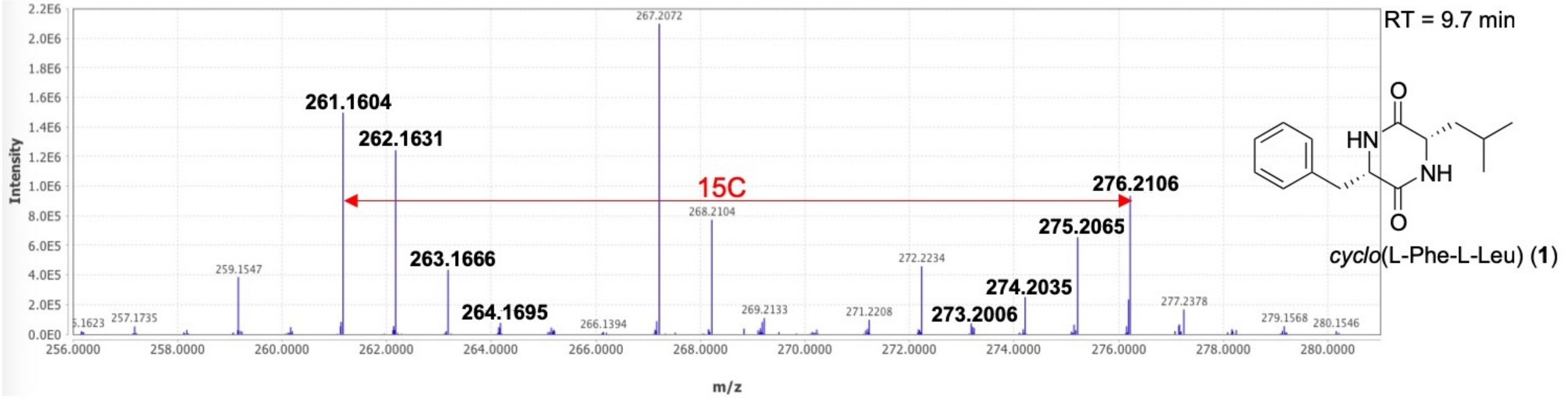

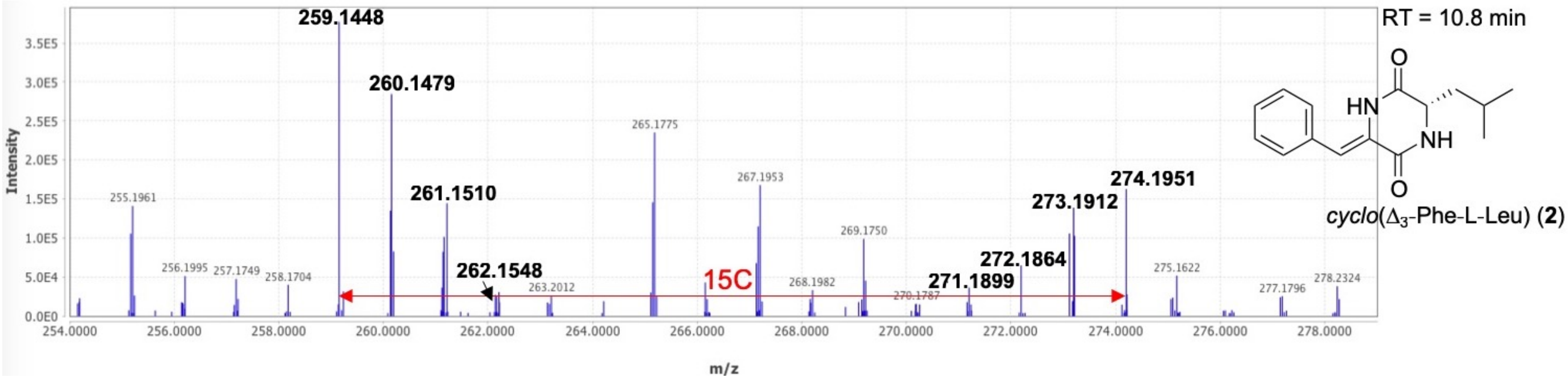

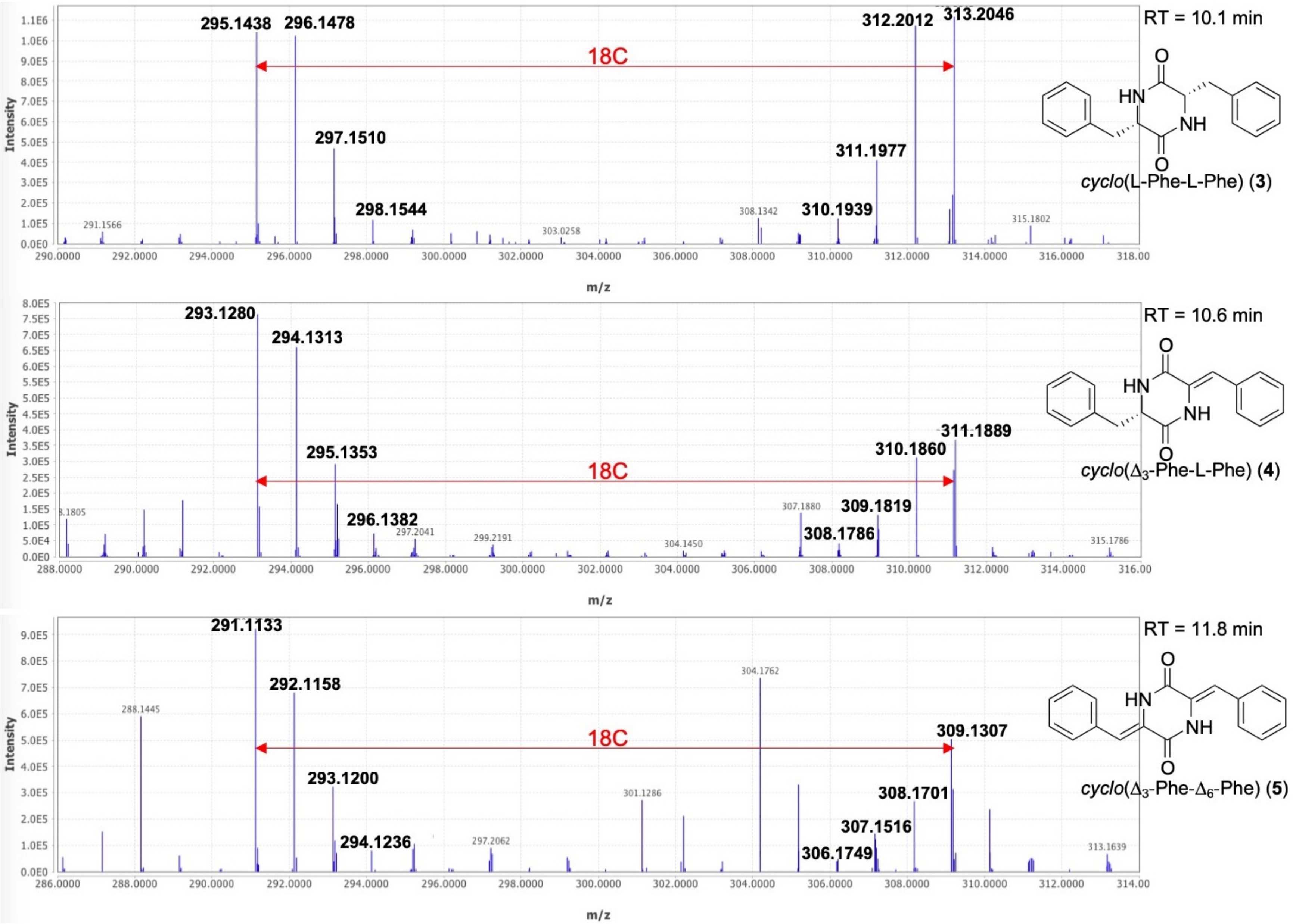

RT = 10.1 min
295.1438
296.1478
297.1510
298.1544
291.1566
303.0258
308.1342
310.1939
311.1977
312.2012
313.2046
315.1802
18C
Intensity
m/z
O
HN
NH
O
cyclo(L-Phe-L-Phe) (3)
RT = 10.6 min
293.1280
294.1313
295.1353
296.1382
297.2041
299.2191
304.1450
307.1880
308.1786
309.1819
310.1860
311.1889
315.1786
18C
Intensity
m/z
cyclo(Δ3-Phe-L-Phe) (4)
RT = 11.8 min
288.1445
291.1133
292.1158
293.1200
294.1236
297.2062
301.1286
304.1762
306.1749
307.1516
308.1701
309.1307
313.1639
18C
Intensity
m/z
cyclo(Δ3-Phe-Δ6-Phe) (5)

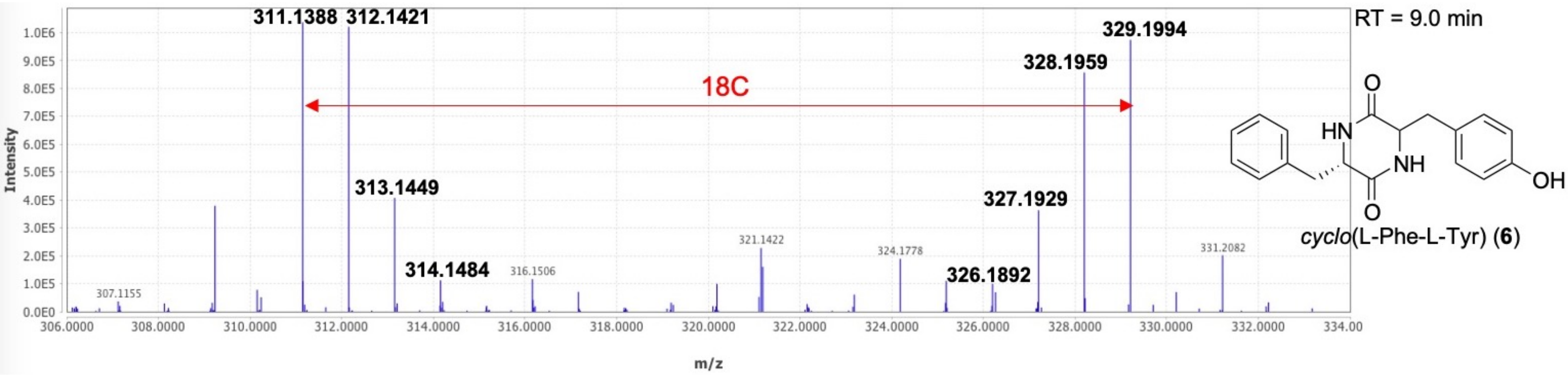
RT = 9.0 min
311.1388
312.1421
313.1449
314.1484
316.1506
307.1155
321.1422
324.1778
326.1892
327.1929
328.1959
329.1994
331.2082
18C
Intensity
m/z
HN
NH
O
O
OH
cyclo(L-Phe-L-Tyr) (6)

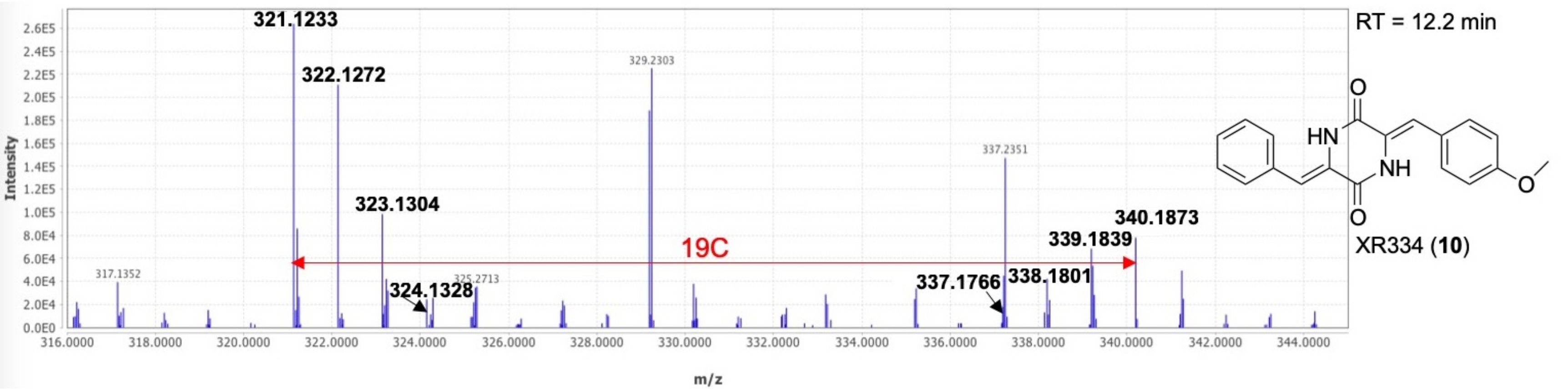
RT = 12.2 min
321.1233
322.1272
323.1304
324.1328
325.2713
317.1352
329.2303
337.2351
337.1766
338.1801
339.1839
340.1873
19C
Intensity
m/z
HN
NH
O
O
O
XR334 (10)

Table S4. Summary of IROA LC/MS data for 40 metabolites most strongly distinguishing *N. dassonvillei* bipyridyl treatment (T12C) from no bipyridyl control (C12C) groups based on RF MDA. Molecular formulae were proposed based on correspondence of experimental exact masses with theoretical formulae containing the number of carbon atoms supported by IROA peak pair evaluation. Compound identities were proposed based on correspondence of LC/MS data between *N. dassonvillei* metabolites and a standard library. Experimental exact masses indicate *m/z*'s for proposed $[M+H]^+$ ^{12}C molecular ions except for entries denoted by *, which indicate proposed $[M+Na]^+$ ions. Box-whisker plots of relative abundances of these metabolites from T12C and C12C are provided in Fig. S2.

Metabolite ID Code	Proposed formula or name	Experimental exact mass *m/z* for $[M+H]^+$ or $[M+Na]^+$	LC retention time (min)	MDA
SECIM.Pos000663	C4H8O4S	153.0217	0.99	2.80
SECIM.Pos000293	C6H10O2	115.0758	7.53	2.44
SECIM.Pos000595	*cyclo*(Δ_3-Phe-L-Leu) (**2**)	259.1442	10.78	2.43
SECIM.Pos000380	C11H15NO5	242.1017	8.13	2.38
SECIM.Pos000264	C10H4NO	154.0338	7.21	2.22
SECIM.Pos000097	C4H7O8P	214.9973	1.44	2.21
SECIM.Pos001017	C14H20O2	221.1538	14.39	2.20
SECIM.Pos000928	C6H11N4O	156.1022	8.28	2.19
SECIM.Pos001384	C9H14N2	151.1235	7.64	2.18
SECIM.Pos000597	C10H18O2	171.1381	10.92	2.18
SECIM.Pos000833	C17H20NP	293.1281 *	10.55	2.00
SECIM.Pos000770	C10H11NO4	210.0767	8.46	1.99
SECIM.Pos000546	C16H24N4O7	385.1761	9.82	1.99
SECIM.Pos000736	C16H6N9O	171.0441	7.64	1.98
SECIM.Pos000052	C6H12O5S	197.048	0.99	1.98
SECIM.Pos000618	C9H15N3O4	230.1173	11.45	1.97
SECIM.Pos000372	C11H18N5O2	253.1521	8.06	1.97
SECIM.Pos000723	C11H11NO2	190.0862	7.42	1.97
SECIM.Pos000116	2-aminophenol	110.0605	1.98	1.96
SECIM.Pos000261	C10H13NO5	228.0864	7.21	1.96
SECIM.Pos001436	C16H23NO3	278.1748	11.64	1.96
SECIM.Pos000311	C8H9NO2	152.0707	7.64	1.96
SECIM.Pos000355	C10H7NO2	196.0371	7.98	1.96
SECIM.Pos000952	C9H11N4O2	208.0967	8.83	1.96
SECIM.Pos001105	L-phenylalanine	188.0681 *	6.23	1.96
SECIM.Pos001284	C10H6N6O7	162.02	7.21	1.96
SECIM.Pos001581	C6H8N3O2S	187.0397	5.87	1.94
SECIM.Pos000900	C7H7N4OS	196.0431	7.37	1.94
SECIM.Pos001324	C11H11NO3	206.0813	8.63	1.94
SECIM.Pos001433	C18H33NO3	312.2529	11.5	1.94
SECIM.Pos000115	pyridoxal	168.0657	1.96	1.93
SECIM.Pos000505	C11H11N3O2S	249.0557	9.34	1.93
SECIM.Pos001059	4-guanidinobutanoic acid	168.0744 *	1.23	1.93
SECIM.Pos001727	C10H14N5OS	253.1015	7.96	1.93
SECIM.Pos000092	uracil	113.0351	1.44	1.91
SECIM.Pos000125	L-isoleucine	132.1024	2.5	1.91
SECIM.Pos000354	C10H7NO2	174.0551	7.98	1.91
SECIM.Pos000508	C9H12O3	169.0863	9.37	1.91
SECIM.Pos000317	C8H16N2O4P2	134.0364	7.65	1.90
SECIM.Pos000898	C14H20N2O5S	329.1167	7.35	1.89
